# Hybrid perfect metamaterial absorber for microwave spin rectification applications

**DOI:** 10.1038/s41598-020-76090-6

**Published:** 2020-11-06

**Authors:** Jie Qian, Peng Gou, Hong Pan, Liping Zhu, Y. S. Gui, C.-M. Hu, Zhenghua An

**Affiliations:** 1 State Key Laboratory of Surface Physics, Institute of Nanoelectronic Devices and Quantum Computing, Key Laboratory of Micro and Nano Photonic Structures (Ministry of Education), Department of Physics, Fudan University, Shanghai, 200433 China; 2 Department of Physics and Astronomy, University of Manitoba, Winnipeg, R3T 2N2 Canada; 3 Collaborative Innovation Center of Advanced Microstructures, Nanjing, 210093 China; 4 Shanghai Qi Zhi Institute, 41th Floor, AI Tower, No. 701 Yunjin Road, Xuhui District, Shanghai, 200232 China

**Keywords:** Applied physics, Condensed-matter physics

## Abstract

Metamaterials provide compelling capabilities to manipulate electromagnetic waves beyond the natural materials and can dramatically enhance both their electric and magnetic fields. The enhanced magnetic fields, however, are far less utilized than the electric counterparts, despite their great potential in spintronics. In this work, we propose and experimentally demonstrate a hybrid perfect metamaterial absorbers which combine the artificial metal/insulator/metal (MIM) metamaterial with the natural ferromagnetic material permalloy (Py) and realize remarkably larger spin rectification effect. Magnetic hot spot of the MIM metamaterial improves considerably electromagnetic coupling with spins in the embedded Py stripes. With the whole hybridized structure being optimized based on coupled-mode theory, perfect absorption condition is approached and an approximately 190-fold enhancement of spin-rectifying photovoltage is experimentally demonstrated at the ferromagnetic resonance at 7.1 GHz. Our work provides an innovative solution to harvest microwave energy for spintronic applications, and opens the door to hybridized magnetism from artificial and natural magnetic materials for emergent applications such as efficient optospintronics, magnonic metamaterials and wireless energy transfer.

## Introduction

Metamaterials offer a great avenue to control the absorption, reflection and transmission of the electromagnetic wave over a wide spectrum range from microwave to visible light^1–5^, which lead to various cutting-edge research directions such as transformation optics^6,7^ and light-matter interaction^8,9^. A typical example of optimized light-matter interaction is perfect absorption of electromagnetic wave with unity energy absorption efficiency and hence facilitating numerous high performance functional devices including plasmonic sensor^10–13^, filter^14,15^, bolometer^16,17^, electromagnetic wave cloaking^18,19^, multi-band/broad-band perfect absorber^20–24^, photodetectors^25–27^ and solar cells^28^. In most of reported perfect metamaterial absorbers (PMAs) so far, however, the enhanced absorption is mainly based on enhanced electric field components and the electric response of the constitution materials including metal ohmic loss and/or dielectric absorption. The metal ohmic loss eventually leads to heat generation which can thereby be utilized in hot carrier related solar-thermal and chemical processes^29^, while the improved dielectric absorption can be implemented in optoelectronic applications such as photodetectors, lasers^30^ and photovoltaic solar cells^31^. Nevertheless, the counterpart magnetic field components are far less addressed and utilized^32^, despite urgent demands in spintronics due to intrinsically weak light-spin interaction strength^33–35^.

In the field of spintronics, manipulation of spin degree of freedom offers tremendous opportunities for future information processing and nonvolatile information storage. The energy utilization of spintronic devices, however, is severely restricted by weak interaction between electromagnetic waves with spins in natural magnetic materials^36–39^. The high frequent spintronic applications therefore require tremendous efforts to overcome this limitation. As an example of high frequent spintronic devices, spin dynamo^40^ has aroused considerable interest due to its unique ability to transform microwave electromagnetic energy to direct current (DC)^41^ and therefore provides a potential solution to realize distributed power system for future nanoelectronics. The reported works^42,43^, however, relied on coaxial cable connection with mandatory transmission line design and demonstrated rather low conversion efficiencies from microwave power to DC power. Therefore, enhanced light-spin interaction is highly demanded to boost the microwave excitation efficiency of spins dynamics and even enable wireless version for real application. As mentioned above, designing artificial metamaterials specifically for spintronic device may be a good choice to satisfy this demand.

In this work, we propose a hybrid perfect metamaterial absorber (HPMA) embedded with ferromagnetic permalloy (Py) to enhance considerably light-spin interaction. The metal/insulator/metal (MIM) tri-layer structure of the HPMA harvests microwave energy and thereby enhances the magnetic fields to excite the spin precession in Py. By optimizing the parameters of the MIM structure to equalize the absorptive and radiative factors of the HPMA, a perfect absorption condition is approached and spin-rectifying DC photovoltage from the embedded Py is substantially enhanced. Without losing generality, our work can be extended to more spintronic applications such giant magnetoresistance (GMR), colossal magnetoresistance (CMR), spin pumping and spin-hall effects^42,44–46^, and dramatically increase their energy conversion efficiency. More functional spintronic devices can therefore be triggered by the hybrid magnetism from artificial and natural magnetic materials, as demonstrated here.

## Results

### Structure and simulations

The HPMA consists of a top copper metal layer with split-ring resonator (SRR) array, a bottom continuous aluminum (Al) foil mirror layer and a sandwiched dielectric layer in which Py stripe is inserted.

**Figure 1:**
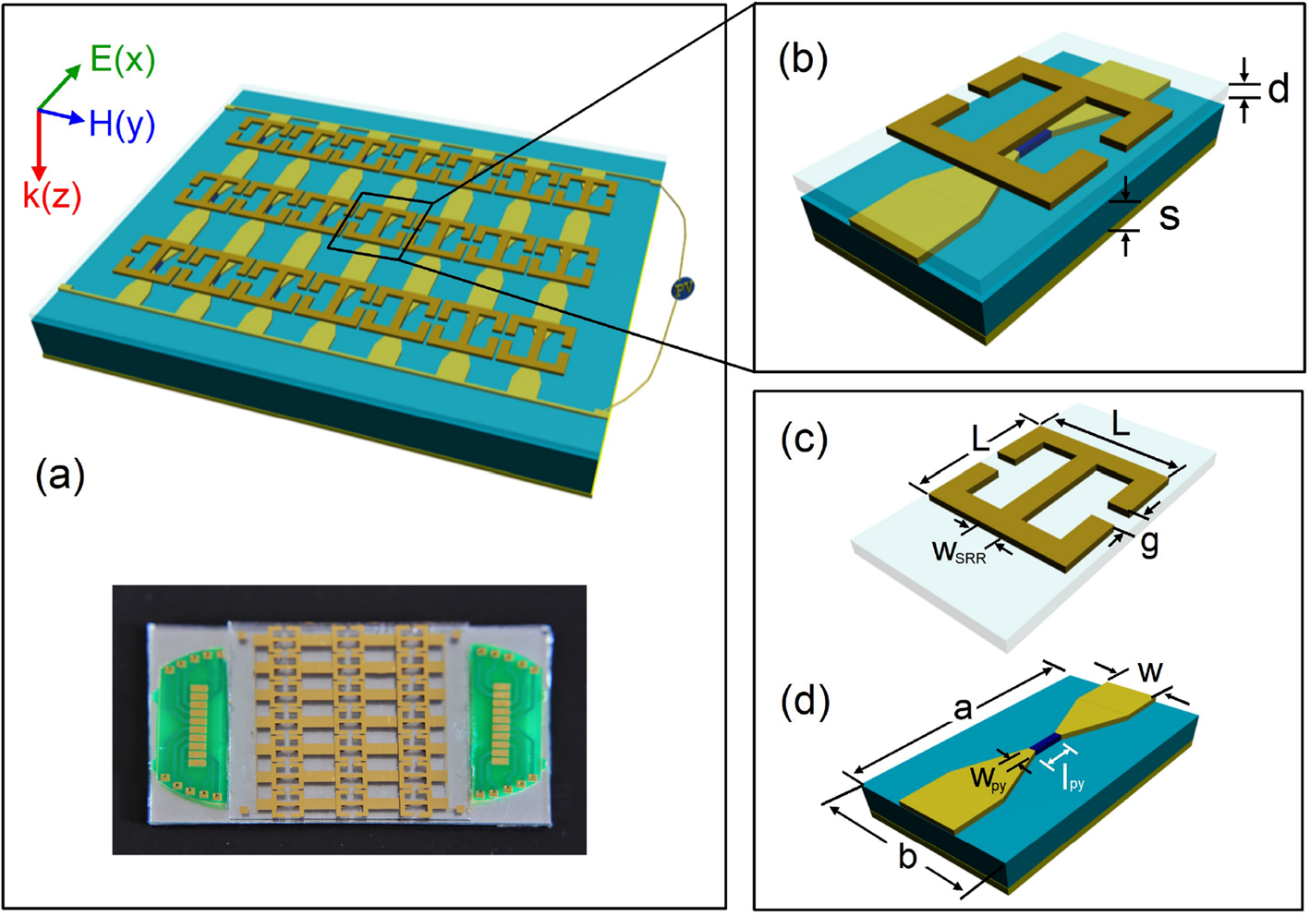
(**a**) A schematic diagram (top) and photoimage (bottom) of the proposed HPMA structure with $3\times 7$ SRR array. (**b**) A unit cell of the HPMA structure. It contains: top metal SRR; Insulator (PET substrate, spin dynamo device or Py stripe, Glass substrate); Metal (Al foil as ground plate). (**c**) A magnified image of SRR on PET substrate with a dimension L = 4.2 mm, $\textsf {w}_{\textsf {SRR}}=0.2\,\hbox {mm}$, g = 0.4 mm, d = 0.55 mm. (**d**) The magnified image of spin dynamo device on glass substrate with the thickness of cavity S, the dimension of substrate and gold electrode is a = 8 mm, b = 4.2 mm, w = 1.7 mm, and the dimension of Py is $\textsf {l}_{\textsf {Py}}=600 \mu \,\hbox {m}$, $\textsf {w}_{\textsf {Py}}=20\mu \,\hbox {m}$, $\textsf {d}_{\textsf {Py}}=45\,\hbox {nm}$ in thick.

Figure 1a schematic shows the schematic view of the $3\times 7$ array HPMA structure: the top layer contains SRRs with periods of $\textsf {L}\times \textsf {L}$ in the $x-y$ plane and the width of SRR is $\textsf {w}_{\textsf {SRR}}$ with a gap g, as shown in Fig. 1c. Different from the concentric SRR^47^, this symmetric SRR structure will function as a localized particle which construct a purely electrical resonant response^48^, as a result the microwave magnetic field can be confined around the central line of the SRR and converted to DC power through the under placed spin dynamo. The sandwiched dielectric layer is a glass or polyethylene terephthalate (PET) substrate with the thickness S on which the Py stripe (act as the spin dynamo) with the length $\textsf {l}_{\textsf {Py}}$ and the width $\textsf {w}_{\textsf {Py}}$ is connected by gold electrodes with a width w, as shown in Fig. 1d. To electrically isolate the magnetic Py layer from top SRR layer, the SRR array is fabricated on another glass substrate with the thickness d and then mechanically attached onto the Py. The total thickness of cavity dielectric spacing layer is therefore the sum of d and S (Fig. 1b). For simplicity, we keep d = 0.55 mm fixed in this work to highlight the cavity-resonance enhancement effect. The bottom mirror layer is a continuous Al foil with a thickness of 100 mm which is thick enough to block all the transmission, i.e., T = 0, so that required one-port configuration for ideally perfect absorption can be reached^49^. The radiative loss of this configuration can be controllable by changing S, thus realizing the perfect absorption conditions, on which the energy that should be dissipated into heat can be almost completely converted into electrical energy in spin dynamo.

**Figure 2:**
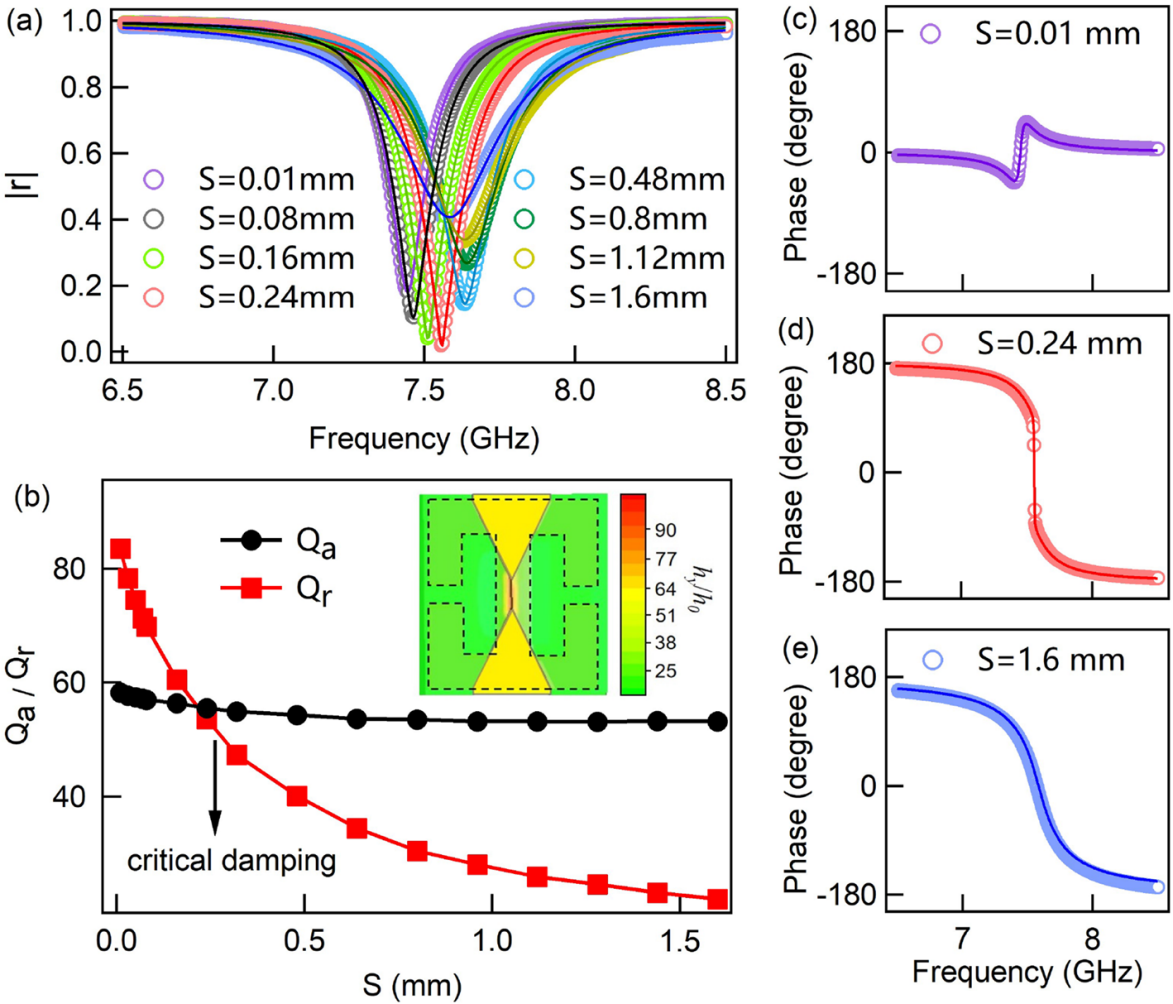
(**a**) Simulations (open circles) and calculations (solid lines) for the reflection amplitude |*r*| of HPMA structure at different dielectric thickness S. (**b**) The absorptive factor $Q_a$ and radiative factor $Q_b$ with versus S obtained by CMT-fitting. The inset displays the enhancement of microwave magnetic field distribution on Py stripe position at resonant frequency of the HPMA. The simulated (open circles) and calculated (solid lines) phase results at (**c**) S = 0.01 mm, (**d**) S = 0.24 mm, and (**e**) S = 1.6 mm corresponding to over damping, critical damping and under damping, respectively.

To simulate and optimize the HPMA structure, the top copper metal, bottom Al metal and the gold electrode for Py are set to be Drude-type dispersion and the dielectric constant of glass substrate is set to be $\varepsilon _{glass}=3.2$ with a constant loss of $\tan \delta _{glass}=0.025$, and the PET substrate $\varepsilon _{PET}=4.5$, $\tan \delta _{PET}=0.002$. The normally incident microwave excites the samples along *z*-direction with its electric/magnetic-field polarization being along *x*/*y*-direction, as schematically shown in Fig. 1a. Simulations are carried out with the three-dimensional finite difference time domain (FDTD) method. The inset in Fig. 2b shows the simulated *y*-component enhancement of microwave magnetic field distribution ($h_y/h_0$, $h_0$ is the microwave magnetic field on the input port) on the position of Py stripe at the resonant frequency, which is enhanced nearly 100-fold with the help of SRR and the bottom Al mirror layer. Open circles in Fig. 2a are the typical simulated spectra of reflection amplitude at different thickness of dielectric layer S, while other parameters are fixed: L = 4.2 mm, $\textsf {w}_{\textsf {SRR}}=0.2\,\hbox {mm}$, g = 0.4 mm, d = 0.55 mm, a = 8 mm, b = 4.2 mm, w = 1.7 mm. From that, with the increasing of S, the reflection amplitude at resonance first decreases to approaching zero at S = 0.24 mm and then increases when $\textsf {S} \geqslant 0.24\,\hbox {mm}$, despite a slight shifts of resonant frequency and bandwidth. Accordingly, the reflection phase varies and only occupies a small range less than $180^\circ$ in the range of $0.1\,\hbox {mm}\leqslant \textsf {S}\leqslant 0.16\,\hbox {mm}$ which implies an over damping region, on the other hand, covers the full $360^\circ$ range in the range of $\textsf {S}\geqslant 0.24\,\hbox {mm}$ which implies an under damping region. The non-monotonous reflection amplitude change and the abrupt phase jump indicate a critical damping or equivalently perfect absorption condition occurs in the range of $0.16\, \hbox {mm}\leqslant \textsf {S}\leqslant 0.24\,\hbox {mm}$. Figure 2c–e display the three typical reflection phases at S = 0.01 mm (over damping), S = 0.24 mm (critical damping), and S = 1.6 mm (under damping), respectively. To be more quantitative, couple-mode theory (CMT) was employed to study these spectra of the simulated HPMA structures. For one-port resonator system, the reflection coefficient *r* can be described as follow^32^.

1 $$ r=-1+\frac{\frac{2}{\tau _r}}{-i(\omega -\omega _0)+\frac{1}{\tau _a}+\frac{1}{\tau _r}} . $$

where $\tau _a$ and $\tau _r$ denote the lifetimes of the resonance due to absorption inside the structure and radiation to the far field, respectively. The solid lines in Fig. 2a,c–d are calculated by Eq. (1) which are well accord with the simulated results. Then we define two quality factors to be $Q_a=\omega _0\tau _a/2$ (the absorptive quality factor) and $Q_r=\omega _0\tau _r/2$ (the radiative quality factor), where $\omega _0$ is the resonant frequency. Hence the absorption of the HPMA at resonance can be described as follow

2 $$ A\left( \omega _0\right) =1-|r\left( \omega _0\right) |^2=\frac{4Q_aQ_r}{\left( Q_a+Q_r\right) ^2} . $$

The absorptive quality factor $Q_a$ and the radiative quality factor $Q_r$ as a function of S are displayed in Fig. 2b. It indicates that the increasing distance between SRR and bottom metal layer would reduce the electromagnetic coupling thus $Q_r$ dramatically decreases, while $Q_a$ of the HPMA structure is insensitive to S. From Eq. (2), it is worth to note that, the intrinsic loss of glass is necessary because it affects $Q_a$ and therefore the perfect absorption condition, i.e., $A(\omega _0)=1$, can be reached only when $Q_a=Q_r$. As shown in Fig. 2b, transition from over damping region $(Q_r>Q_a)$ to under damping region $(Q_r<Q_a)$ occurs when S increases in the range of $0<\textsf {S}\leqslant 1.6\,\hbox {mm}$, and the perfect absorption condition (critical damping) takes place near S = 0.24 mm.

### Spin rectification near perfect absorption condition

**Figure 3:**
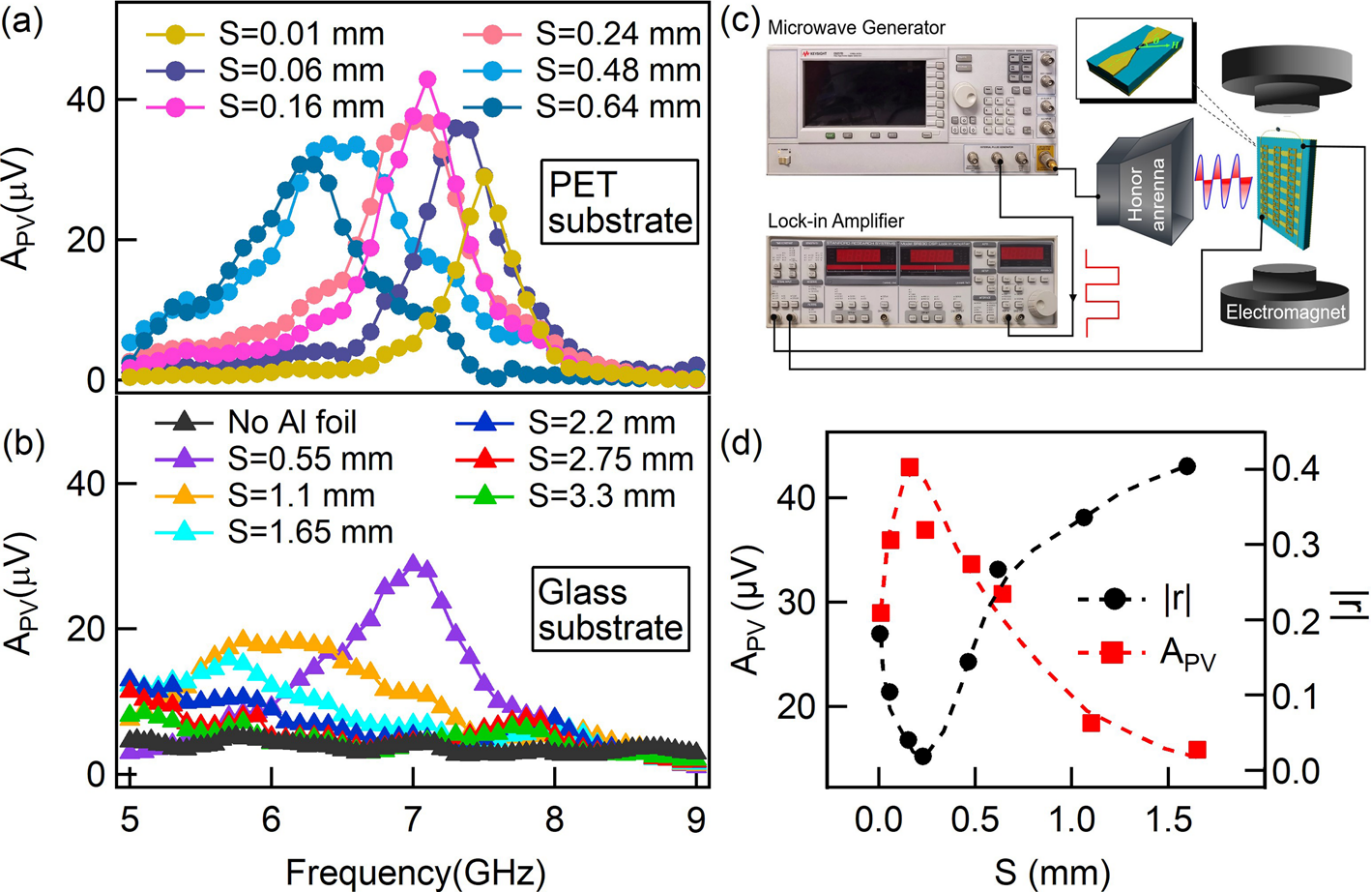
The amplitude of photovoltage $A_{PV}$ induced by spin rectification effect with different dielectric thicknesses S: (**a**) PET substrate with a thickness of 0.01 mm to 0.64 mm. **(b)** Glass substrate with a thickness of 0.55 mm to 3.3 mm and the condition without bottom Al mirror. (**c**) The schematic of the experiment setup, inset is the diagram of the angle $\theta$ between static magnetic field and Py stripe. (**d**) The photovoltage amplitude $A_{PV}$ (left axis) and simulated reflection amplitude |*r*| (right axis) versus S, dash lines are guide lines.

**Figure 4:**
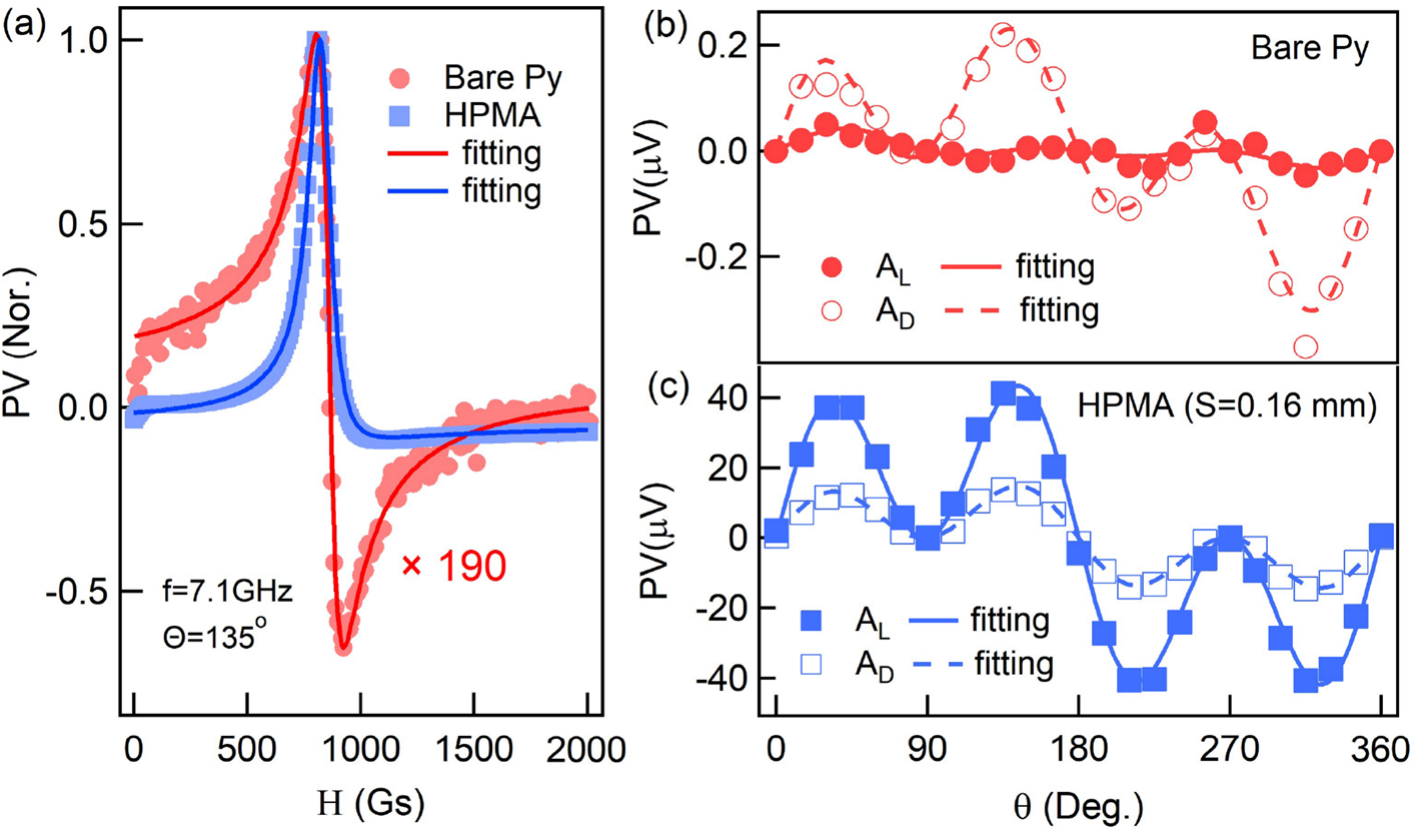
(**a**) The normalized line shape of FMR for the Bare Py (red circles) and HPMA (blue squares) configurations at resonant frequency f = 7.1 GHz with $\theta =135^\circ$, solid lines are corresponding fitted results. The Lorenz $A_L$ (solid symbols) and Dispersive components $A_D$ (open symbols) as function of $\theta$ with respect to (**b**) the Bare Py and (**c**) the HPMA configuration at f = 7.1 GHz. Solid and dash lines are corresponding fitted results.

Under the guidance of simulation, we experimentally placed Py stripes under the top SRRs structure with the distance of d and each stripe is aligned to the central arm of SRR, which enables adequately utilization for the magnetic field component of the HPMA resonant structures. The magnetic field induced by the resonantly oscillating current in the central arm of each SRR excites the spin precession in underneath Py stripe. Begin to measure the spin rectifying signal, we applied a static magnetic field in the $x-y$ plane with an angle of $\theta =135^\circ$ with respect to the Py strip (*x*-direction). This static magnetic field breaks the symmetry of the two electrodes for each Py stripe such that anisotropic magnetoresistance (AMR) effect^50^ comes into play and the motion of spin-polarized charges becomes asymmetric along $+x$ and $-x$ directions. Eventually a non-zero rectifying signal can be generated between two electrodes under microwave excitation, enabling the functional spin dynamo^50^. Using the experiment setup as shown in Fig. 3c, the photovoltage induced by spin rectification was detected and it can be described as $PV\propto \langle j\cdot m\rangle$, where *j* is the microwave current induced by the microwave electric field in Py stripe, and *m* is the non-equilibrium magnetization driven by the microwave *h*-field, and $\langle \rangle$ denotes the time average. The photovoltage signal near ferromagnetic resonance (FMR) can be derived as

3 $$ PV=A_L\frac{\Delta H^2}{(H-H_0)^2+\Delta H^2}+A_D\frac{\Delta H\left( H-H_0\right) }{\left( H-H_0\right) ^2+\Delta H^2} . $$

where $A_L$ and $A_D$ are the amplitudes for the Lorentz and Dispersive components, respectively, $\Delta H$ is the linewidth and $H_0$ is the resonant magnetic field for FMR. Taking account of both Lorentz and Dispersive contributions, the amplitude of the photovoltage at the FMR can be defined to be $A_{PV}=\sqrt{A_L^2+A_D^2}$. In experiments, to have a wide range coverage for the thickness S, two different substrates, i.e., PET (0.01 mm thick/piece) and Glass (0.55 mm thick/piece) were used. Figure 3a,b show the spin rectification results, $A_{PV}$ for a thinner thickness of cavity with PET substrate and for a thicker thickness of cavity with glass substrate, respectively. From that, $A_{PV}$ reaches a maximum value at S = 0.16 mm, implying being close to perfect absorption condition. This value agrees reasonably with simulation in Fig. 2, taking account that PET has a larger permittivity $(\varepsilon _{PET}=4.5)$ than glass $(\varepsilon _{glass}=3.2)$. To be more clearly, both the experimental $A_{PV}$ and simulated |*r*| with different S of the HPMA are displayed in Fig. 3d. From that, $A_{PV}$ indeed exhibits a peak at a position being consistent with the position that minimum reflection occurs. Comparing $A_{PV}$ at S = 0.16 mm with that in Bare Py, our HPMA structure enables $A_{PV}$ achieves approximately 190-fold enhancement on the perfect absorption condition, as analyzed from Fig. 4. We mention that the experimentally observed enhancement $(\sim \,190)$ of spin-rectifying photovoltage arises from the net contribution of both microwave magnetic field ($h_y$, $\sim \,100$-fold enhancement) and electric field ($e_x$, $\sim \,1.9$-fold enhancement) induces microwave current $j_x$ in Py, in which the low electric field enhancement here is screened by the central arm of the SRR structure due to a small distance between Py and SRR. It is therefore evident that the enhancement of spin-rectifying photovoltage originates from the improved and nearly perfect absorption of the whole structure and thereby induced magnetic field enhancement. Further enhancement of the spin-rectifying dynamo performance can be realized by utilizing both electric and magnetic field enhancement^35^.

Further more, the relative phase between the microwave magnetic field and the microwave current (or the electric field $e_x$) on the perfect absorption condition (S = 0.16 mm) can be confirmed by analyzing the Lorenz and Dispersive components. Considering $h_y\gg h_x$, $h_z$ in the measurement configuration, these two components is approximated to the following relationship

4 $$ A_L\approx -\frac{\Delta R\cdot j_x\cdot A_{yy}\cdot h_y}{2M_0}\sin (2\theta )\cos (\theta )\sin (\Phi _y) . $$

5 $$ A_D\approx -\frac{\Delta R\cdot j_x\cdot A_{yy}\cdot h_y}{2M_0}\sin (2\theta )\cos (\theta )\cos (\Phi _y) . $$

where $\Delta R$ is the resistance change induced by AMR effect, $j_x$ is the current excited by microwave electric field along Py strip, $A_{yy}$ is real number related to the properties of Py, $\Phi _y$ is the relative phase between magnetic field and electric field in *y*-direction. $A_L$ and $A_D$ determines the line shape of FMR, as shown in Fig. 4a, red circles display the antisymmetric line shape of FMR, due to the photovoltage in Bare Py case is dominated by $A_D$, and the blue squares display the nearly symmetric line shape which because $A_L$ is dominates the photovoltage in the HPMA case. The normalized photovoltages shown in Fig. 4a are measured at resonant frequency of HPMA (f = 7.1 GHz) with $\theta =135^\circ$, and the solid red line and blue line are fitted results corresponding to Bare Py case and the HPMA case by using Eq. (3). Actually, as predicted in Eqs. (4) and (5), the relative phase $\Phi _y$ between electric and magnetic field can be extracted by measuring angular-dependent photovoltages. Figure 4b,c show the angular-dependent $A_L$ and $A_D$ components in Bare Py case and the HPMA case, respectively, the measurements are plotted as open/solid symbols and the calculations from Eqs. (4) and (5) are plotted as solid/dash lines. Numerical fitting results show $\Phi _y=6.37^\circ$ for bare condition and $\Phi _y=71.5^\circ$ for HPMA condition. Theoretically, the electromagnetic filed in bare condition is plane wave and thus $\Phi _y=0$; while in case of the HPMA, it would induce a phase difference $\Phi _y=90^\circ$ between the electric and magnetic fields if it purely from the resonant SRR structure. The experimentally derived values sensed by Py stripes $\Phi _y=6.37^\circ$ for Bare Py condition and $\Phi _y=71.5^\circ$ for the HPMA condition are very close to the theoretical expectation, which indicate that the HPMA resonance indeed plays a critical role in the enhanced spin dynamo signal.

## Discussion

In conclusion, we propose and demonstrate a novel HPMA structure that harvests wireless microwave energy and generates direct current based on spin rectification. Perfect absorption condition enhances the spin dynamo signal by an approximate factor of 190-fold due to mainly enhanced microwave magnetic field of the SRR resonance. Although the enhanced spin-rectifying signal demonstrated here is in microwave range, the metamaterial cavity structure can be scaled and expanded to wider frequency bands. Our work paves a way to utilize hybrid natural and artificial magnetism to realize functional devices such as high frequent spintronics, magnonic metamaterials and wireless energy converters etc.

## Methods

### Simulations

Numerical simulation is performed by the finite difference time domain method, CONCERTO 7.0, Vector Fields Limited, England (2008).

### Sample fabrication

All the samples in this experiment are fabricated by standard optical lithography and magnetron sputtering methods.

### Spin rectification measurement setup

To measure the spin rectification photovoltage, we applied a static magnetic field in the $x-y$ plane with an angle of $\theta$ with respect to the Py strip (*x*-direction). A standard microwave generator (Agilent E8257D) whose amplitude was modulated by a square wave with a period 120 $\mu$s and a pulse width of 60 $\mu$s, emitted the 5–9 GHz microwave onto the sample through an honour antenna. The polarization of the microwave was along the *x*-direction. The photovoltage generated by Py spin dynamo was detected by a lock-in amplifier (Stanford SR830) with sweeping the external DC magnetic field in the range of $-2000$ Gs $\sim \,+2000$ Gs. All the measurements were carried out at room temperature. The schematic diagram of the experiment setup is shown in Fig. 3c.

## Data Availability

The data that support the findings of this study are available from the corresponding author upon reasonable request.
